# Synthesis, Characterization, and Biodegradability of a Superabsorbent Cellulose-Based Hydrogel Derived from Olive Pomace

**DOI:** 10.3390/polym18202495

**Published:** 2026-10-12

**Authors:** Sukru Esin, Mustafa Degirmenci, Eyyup Karaogul, Achraf Berradi, Ahlam Lafdali, Faissal Aziz, Pınar Küce Cevik, Salih Aydemir

**Affiliations:** 1 Department of Soil Science and Plant Nutrition, Faculty of Agriculture, Harran University, Sanliurfa 63290, Türkiye; sukru.esin@gopa.eu (S.E.); aydemirsalih15@gmail.com (S.A.); 2 Department of Chemistry, Faculty of Science, Harran University, Sanliurfa 63290, Türkiye; 3 Department of Industrial Engineering, Faculty of Engineering, Harran University, Sanliurfa 63290, Türkiye; eyyupkaraogul@gmail.com; 4 Water Sciences, Microbial Biotechnologies and Natural Resources Sustainability Laboratory, Faculty of Sciences Semlalia, Cadi Ayyad University, Marrakech 40000, Morocco; achraf97berradi@gmail.com (A.B.); lafdaliahlam99@gmail.com (A.L.); 5 Kokand University, 28-A Turkiston Street, Kokand 150700, Fergana Region, Uzbekistan; 6 Department of Molecular Biology and Genetics, Faculty of Science, Harran University, Sanliurfa 63290, Türkiye; pinarkcvk@harran.edu.tr

**Keywords:** olive pomace, carboxymethyl cellulose, epichlorohydrin cross-linking, hydrogel, swelling behavior, water retention, soil-burial biodegradation

## Abstract

In this study, a cellulose-based hydrogel was derived from olive pomace through a three-stage valorization process. In the first stage, cellulose was extracted from de-oiled olive pomace, yielding 9.16%. In the second stage, the extracted cellulose was converted to sodium carboxymethyl cellulose (CMC) via carboxymethylation with monochloroacetic acid (MCA) under alkaline conditions. In the third stage, the synthesized CMC was chemically crosslinked with epichlorohydrin (ECH) in an alkaline medium to produce a biobased hydrogel. The resulting material exhibited an amorphous phase fraction of 80.93% and an exceptional equilibrium swelling ratio of 2616% after approximately 12 days of immersion in distilled water. Thermogravimetric analysis (TGA) revealed that the hydrogel exhibited good thermal stability, with an onset degradation temperature of 236.05 °C, and retained 27.05% of its mass at 600 °C. Furthermore, time-dependent deswelling measurements demonstrated strong water-retention performance, with a cumulative mass loss of only 20.93% after 6.5 h under ambient evaporation conditions. The biodegradability test was systematically assessed via a 28-day soil burial test using two different carrier systems: perforated polyethylene bags and muslin/cheesecloth bags. Following 28 days of soil burial, the hydrogel exhibited gravimetric mass losses of 41.25% and 53.88% in the perforated polyethylene and muslin/cheesecloth carrier systems, respectively. These results indicate that the hydrogel derived from olive pomace is an olive pomace-derived biodegradable water-retention material with significant potential for agricultural soil conditioning, controlled-release fertilizer systems, and other environmental applications.

## 1. Introduction

Agricultural production is increasingly constrained by water scarcity, poor soil water-holding capacity, and frequent irrigation requirements, particularly in arid and semi-arid regions. Superabsorbent hydrogels offer a promising solution by absorbing and retaining large amounts of water and gradually releasing it to the soil. Hydrogels can be produced from synthetic or bio-based materials, with increasing interest in renewable and environmentally compatible alternatives [1]. Among the bio-based materials, cellulose and its derivatives are particularly promising for hydrogel development [2]. As is known, cellulose, the most abundant renewable biopolymer on Earth, has garnered immense attention as a sustainable raw material for developing advanced functional materials [3]. Found predominantly in plant cell walls, this biopolymer possesses remarkable attributes including biodegradability, biocompatibility, non-toxicity, environmental friendliness, and low cost, making it an ideal candidate for diverse industrial, biomedical, and environmental applications [4]. Each anhydroglucose unit in the cellulose backbone bears three hydroxyl groups (at C-2, C-3, and C-6 positions), which serve as reactive sites for various chemical modifications such as esterification, etherification, oxidation, and crosslinking [3]. Among the numerous cellulose derivatives, carboxymethyl cellulose (CMC), produced via partial substitution of hydroxyl groups with carboxymethyl groups, is one of the most widely studied water-soluble anionic polyelectrolytes [5]. Introducing carboxymethyl groups enhances hydrophilicity, ion-exchange capacity, and pH responsiveness, making CMC particularly attractive for hydrogel fabrication [6].

Hydrogels are three-dimensional polymeric networks that absorb and retain large quantities of water without dissolving, owing to their hydrophilic functional groups (such as –OH, –COOH, –CONH_2_, and –NH_2_) and crosslinked architecture [5,7]. The unique combination of high water-absorption capacity, soft and rubbery consistency, and biocompatibility has positioned hydrogels as promising materials for applications spanning agriculture [4], drug delivery [8], heavy metal removal [9], and dye adsorption [10]. These materials can be crosslinked physically or chemically. However, chemical crosslinking via covalent bonds generally yields networks with superior structural stability, mechanical properties, and water absorption capacity compared to physically crosslinked counterparts [5].

Among various chemical crosslinking agents, epichlorohydrin (ECH; 1-chloro-2,3-epoxypropane) has been widely used as a crosslinker for preparing cellulose-based hydrogels [3]. For instance, ECH reacts with the hydroxyl groups of CMC under alkaline conditions, forming stable ether (C–O–C) bonds that confer high hydrolytic stability, chemical durability, and permanent network architecture [3]. Additionally, each ECH-crosslink introduces a new hydroxyl group, partially compensating for the consumption of original hydroxyl groups during crosslinking [3]. Washing after hydrogel synthesis effectively eliminates the toxicity of the remaining pure ECH, as confirmed by FTIR and NMR analyses in previous studies [3,5].

An increasing number of studies have reported the development of ECH-crosslinked CMC-based hydrogels for a wide range of applications [11]. Wei et al. [9] developed ECH-crosslinked carboxymethyl cellulose fiber (ECMCF) for rapid cadmium removal, achieving a maximum adsorption capacity of 150.60 mg/g, with equilibrium reached within 2 min. Han et al. [12] prepared CMC-ECH/polyacrylamide (PAM) composite hydrogels with a compressive strength of 70 kPa for Cu(II) removal, achieving a maximum adsorption capacity of 75.93 mg/g and excellent reusability over 5 cycles. Jeong et al. [6] synthesized dual-component hydrogels by crosslinking CMC with carboxymethyl β-cyclodextrin using ECH, achieving an eight-fold increase in storage modulus compared to CMC alone while maintaining high swelling capacity for tetracycline drug delivery. Khushbu et al. [10] fabricated NaALG/ECH/CMC hydrogels for dye removal and reported maximum adsorption capacities of 864.8 mg/g for Brilliant Green and 193.1 mg/g for Safranin-O. Muharam et al. [13] prepared starch-ECH-carboxymethyl cellulose superabsorbent hydrogels from cassava starch, achieving a swelling ratio of 1028.5% upon carboxymethyl functionalization. Kono et al. [8] developed ECH-crosslinked CMC hydrogels for carboplatin delivery, demonstrating pH-dependent swelling, approximately 70% drug release within 12 h, and biocompatibility.

While these studies have significantly advanced the field of CMC-ECH hydrogels, several critical gaps remain. The novelty of this study lies in valorizing olive pomace, an agro-industrial waste, through sequential cellulose extraction, carboxymethylation, and ECH crosslinking to obtain a superabsorbent hydrogel. Unlike most reports that use commercial cellulose or CMC, our approach starts from raw olive pomace. First, the majority of reported CMC-ECH hydrogels use commercially available cellulose or CMC as starting materials, thereby increasing production costs and limiting sustainability [6,8,9,12]. Second, most studies focus primarily on adsorption or drug delivery applications, with relatively limited investigation of water-retention behavior over time and deswelling behavior properties of paramount importance for agricultural and environmental applications [9,10,12]. Third, the systematic evaluation of biodegradation behavior, a critical parameter for environmental applications and end-of-life management, remains largely unexplored in the CMC-ECH hydrogel literature, despite the growing emphasis on waste valorization, bio-based/waste-derived precursor materials. Fourth, the valorization of lignocellulosic agricultural waste streams as cellulose precursors for CMC-ECH hydrogel synthesis remains underexplored, despite the dual environmental benefit of waste utilization and value-added product generation.

Olive pomace, the solid residue remaining after olive oil extraction, represents an abundant and largely underutilized lignocellulosic biomass in Mediterranean countries. According to the FAO’s “Focus on olive oil” report [14], global production is forecast to reach 3.4 million tonnes in the 2025/26 season, and since global olive oil production already exceeds 3 million tons annually, the generation of olive pomace poses significant waste management challenges. The cellulose fraction in olive pomace, though embedded in a complex lignin–hemicellulose matrix, can be isolated by alkaline extraction and subsequently functionalized to CMC for hydrogel synthesis.

In this study, to the best of our knowledge, we report one of the first comprehensive investigations of the synthesis, biodegradation, and characterization of an ECH-crosslinked CMC hydrogel derived from olive pomace waste.

The specific objectives of this work are: (i) to extract cellulose from de-oiled olive pomace through alkaline delignification and convert it to sodium carboxymethyl cellulose via etherification process; (ii) to fabricate a three-dimensional hydrogel network through chemical crosslinking using ECH as a crosslinker under selected alkaline conditions; (iii) to comprehensively characterize the chemical structure, crystalline morphology, thermal stability, and morphological structure using Fourier Transform Infrared Spectroscopy (FTIR), X-ray Diffraction (XRD), Thermogravimetric Analysis (TGA), and Scanning Electron Microscopy (SEM); (iv) to evaluate the swelling behavior, time-dependent swelling profile, and deswelling/water-retention performance of the resulting hydrogel over time; and (v) to assess the biodegradation of the synthesized hydrogel under simulated soil conditions through a 28-day soil burial test using two different carrier systems.

The novelty of the present study lies not in using epichlorohydrin as a CMC cross-linking agent, which is already well established, but in implementing an integrated valorization route for olive pomace. In this approach, olive pomace serves as the starting agro-industrial residue for cellulose recovery, followed by conversion to CMC and subsequent ECH cross-linking to produce a superabsorbent hydrogel. Thus, rather than relying on commercial cellulose or CMC, the present work integrates the successive transformation of an olive-oil-industry residue into a functional water-absorbing material. The resulting hydrogel was systematically characterized for its structural, morphological, thermal, swelling, water-retention, and biodegradation properties. Its swelling performance was also quantitatively compared its swelling performance with previously reported CMC–ECH hydrogels to position the developed material within the existing literature.

## 2. Materials and Methods

### 2.1. Materials

De-oiled dry olive pomace was sourced directly from the Zeymak Pomace Oil Processing Facility located in Nizip, Gaziantep, Turkey. The de-oiled dry olive pomace used in this study was characterized as follows (all values on a dry weight basis): moisture 7.2%, carbohydrate 41.5%, lipid 4.1%, protein 7.8%, ash 8.6%, and dietary fiber 30.8%. Before extraction, the crude biomass was washed several times, filtered, and oven-dried at 60 °C to achieve a constant baseline weight. Sodium hydroxide (NaOH), monochloroacetic acid (MCA), ECH, and ethanol were obtained from Merck (Merck KGaA, Darmstadt, Germany) and used without further purification steps. Distilled water was used across all synthetic and analytical operations.

### 2.2. Quantitative Cellulose Extraction from Olive Pomace Matrix

**Figure 1:**
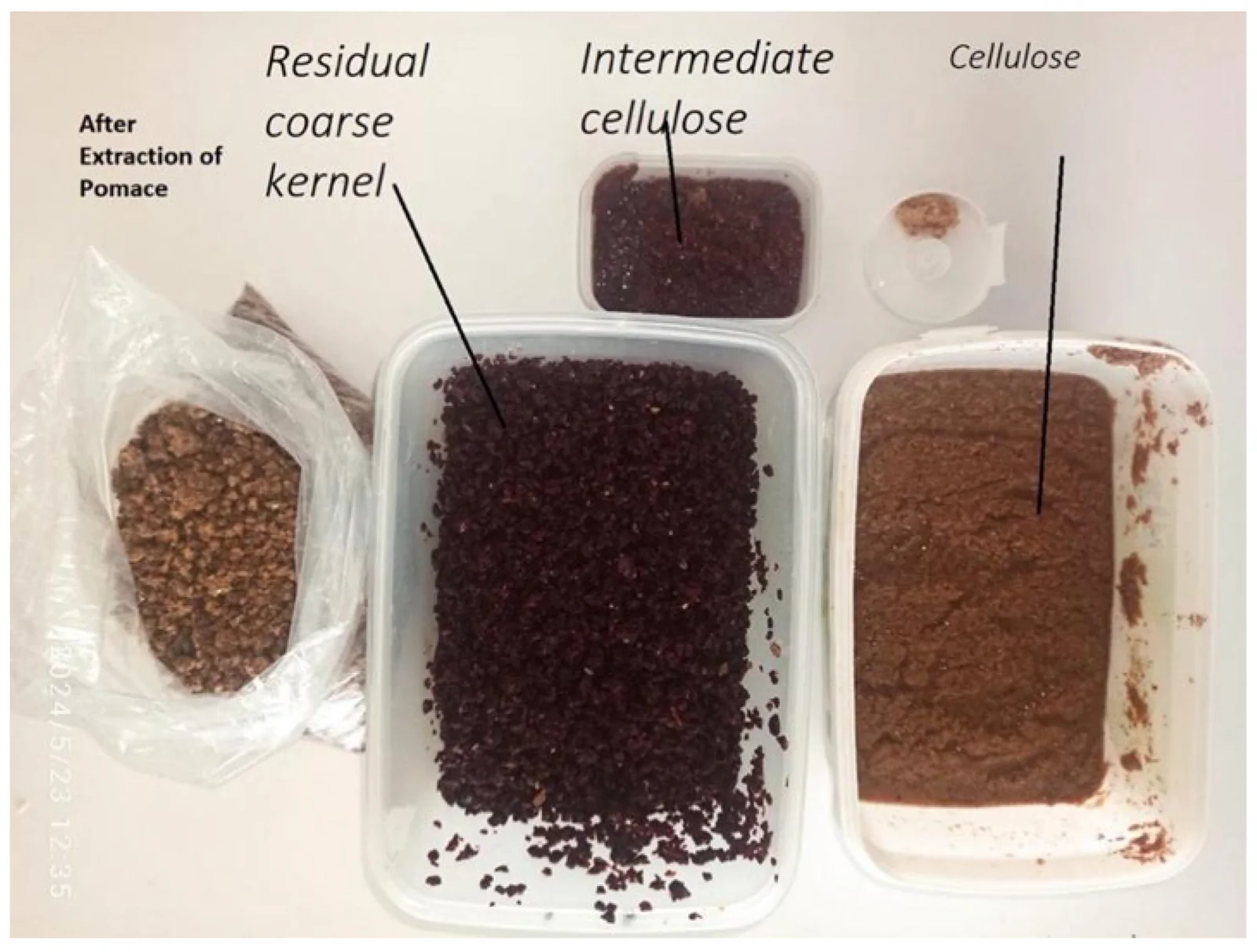
Photographic illustration of isolating the cellulose-rich fraction from de-oiled olive pomace.

To isolate the cellulose fraction from lignin and hemicellulose, an alkaline chemical extraction method was used. A sealed, heavy-gauge pressure vessel capable of safely maintaining temperatures up to 120 °C and pressures up to 2 bar was used. The alkaline extraction was carried out using a 20% (*w*/*v*) NaOH solution for 2 h under the specified high-temperature and high-pressure conditions. A precise starting mass of 410 g of processed, dry, de-oiled olive pomace was introduced into the reaction chamber. After optimizing the alkaline delignification steps, the gravimetric mass yield distribution across all component fractions was determined. Following the alkaline treatment (Figure 1), the solid fraction was separated and repeatedly washed with distilled water. The recovered material was then oven-dried to constant weight, and the masses of the recovered fractions were recorded for gravimetric yield calculations. The bulk of the raw biomass consisted of unreactive, highly dense, coarse endocarp kernels, accounting for 78.10% (320.21 g) of the recovered mass.

The alkaline treatment yielded 37.56 g of a cellulose-rich fraction, corresponding to a 9.16% extraction yield. As no bleaching treatment was performed, the recovered material retained a slight brownish coloration, which may be attributed to residual lignin and other chromophoric components.

Despite this large inert fraction, the alkali treatment successfully isolated 37.56 g of purified cellulose-rich fraction (Figure 1), yielding an absolute extraction efficiency of 9.16%. Additionally, an intermediate cellulose fiber co-product fraction was used with a mass yield of 4.16% (17.07 g). The net unaccounted mass loss throughout the multi-stage separation process was 8.57%. Table 1 presents the mass distribution and yields of the fractions recovered from de-oiled olive pomace. All percentages are expressed on a dry-weight basis relative to the initial dry biomass mass (410 g).

### 2.3. Synthesis and Molecular Weight Measurement of Sodium Carboxymethyl Cellulose (CMC)

The extracted cellulose-rich fraction was reacted with monochloroacetic acid (MCA) to synthesize CMC. It was carried out by adding 4 g of extracted cellulose to 100 mL of 95% (*v*/*v*) ethanol. Next, the solution was alkalized by adding 10 mL of 45% (*w*/*v*) NaOH solution, with constant stirring at room temperature for 30 min. The addition of 5 g of monochloroacetic acid (MCA) was then carried out to the solution. The stirring was continued for 6 h at room temperature, followed by heating at 60 °C for 30 min. After the reaction, the mixture was allowed to cool to room temperature, then gradually neutralized with glacial acetic acid under continuous stirring, without using an ice bath. The neutralized mixture was then filtered. The obtained filtrate (CMC) was washed several times with methanol, filtered, and dried in an oven at 70 °C until constant weight.

The degree of substitution of CMC (DS) was 0.45, and the molecular weight (MW) of 180 kDa was determined according to ASTM D1439 [15] and gel permeation chromatography, respectively, as described by Kamthai et al. [16]. The ASTM D1439 method was followed by adding 1 g of synthesized CMC to 20 mL of a solution mixture (19 mL ethanol and 1 mL HNO_3_) and stirring for 15 min. The acidic CMC was then washed with ethanol and dried at 105 °C for 3 h. Next, 1 g of the dried product was dissolved in an alkaline solution (25 mL of 0.5 N NaOH mixed with 100 mL of distilled water) and titrated with HCl (0.5 N) to a phenolphthalein endpoint. The CMC MW distribution was determined using a Polymer Laboratories PL-GPC 50 Plus (Agilent Technologies, Santa Clara, CA, USA) system equipped with a refractive index detector. The analysis was performed using two PLgel MIXED-C columns (Agilent Technologies, Santa Clara, USA) (5 µm), covering a molar mass range from 200 to 2 × 10^6^ g·mol^−1^, thermostated at 35 °C. CMC samples were dissolved in distilled water, dialyzed, freeze-dried, and then dissolved in THF containing 1% LiBr before filtration and injection into the GPC system. The mobile phase was THF/1% LiBr at 30 °C, with a flow rate of 1.0 mL/min.

The GPC system was calibrated using pullulan standards with nominal molecular weights of 5800, 12,200, 48,800, 107,000, 194,000, and 404,000 Da.

### 2.4. Synthesis of CMC/ECH Hydrogel

CMC/ECH hydrogel was synthesized according to a modified procedure based on previously reported methods [6,17]. Briefly, in a 50 mL round-bottom flask, a solution was prepared by dissolving 0.75 g of NaOH in 10 mL of distilled water. Subsequently, 0.54 g of olive pomace-derived CMC was gradually added to the alkaline solution under continuous mechanical stirring (200–600 rpm) at room temperature to obtain a homogeneous polymer solution. 1.5 mL of ECH was then slowly added to the CMC solution as a crosslinking agent. The reaction mixture was stirred (100 rpm) at room temperature for 20–30 min to ensure uniform dispersion of the cross-linker. Afterward, the reaction flask was transferred to a thermostated oil bath maintained at 50–55 °C, and continuous stirring was maintained at 50 rpm to contribute to crosslinking. Gel formation occurred within approximately 10 min, after which the mechanical stirring was stopped due to the increased viscosity of the reaction mixture. The gel was then kept at 50–55 °C for an additional 2 h to ensure the completion of the cross-linking reaction [6,17].

The obtained hydrogel was cut into uniform pieces and initially immersed in a 50 mL ethanol/water mixture (1:1 (*v*/*v*)) to remove unreacted components and other soluble impurities. Then, the hydrogel was cut into small pieces and washed 3–4 times with distilled water to completely remove residual ECH and NaOH. Finally, the purified CMC/ECH hydrogel was dried in an oven at 50–60 °C until reaching a constant weight.

### 2.5. Characterization

The synthesized hydrogel was characterized using a range of analytical techniques, as described below.

- Fourier-transform infrared analysis: FTIR spectra were recorded using a Shimadzu IRTracer-100 FTIR spectrophotometer (Shimadzu Corporation, Kyoto, Japan) to identify characteristic functional groups and confirm the chemical structure of the extracted cellulose, CMC, and the synthesized hydrogel. FTIR analysis was performed as described by Alam et al. [17], Salleh et al. [4,5]. Each sample was prepared as a pellet by thoroughly mixing 1 mg of it with 99 mg of KBr (Potassium bromide).
- X-ray Diffraction analysis: XRD patterns were recorded using a Rigaku Ultima III diffractometer (Rigaku Corporation, Tokyo, Japan) (Cu Kα, λ = 1.5406 Å) over a 2θ range of 2–90° at a scanning speed of 5°/min. To determine the crystallinity index (CrI), the XRD profiles were deconvoluted into crystalline and amorphous components using Gaussian peak fitting, as described by Karaogul et al. [18]. The CrI was calculated according to the equation:
  CrI (%) = Ac/(Ac + Aa) × 100
  where Ac is the integrated area of the crystalline peaks, and Aa is the area of the amorphous contribution. Crystallite size was estimated using the Scherrer equation, D = Kλ/(βcosθ), where D is the crystallite size, K is the shape factor (0.9), λ is the wavelength, β is the full width at half maximum (FWHM), and θ is the Bragg angle. This approach allowed for quantitative comparison of crystallinity changes after carboxymethylation and crosslinking.
- Scanning Electron Microscopy analysis: SEM images were obtained using a Zeiss Evo 50 (Carl Zeiss AG, Oberkochen, Germany) and a Zeiss & Evo LS 10 (Carl Zeiss AG, Oberkochen, Germany) scanning electron microscope to investigate the morphological characteristics and surface structures of the extracted cellulose, CMC, and crosslinked hydrogel. SEM imaging was conducted as in Alam et al. [17] and Jeong et al. [6]. This analysis was performed to evaluate changes in morphology and the development of the porous network structure resulting from carboxymethylation and epichlorohydrin crosslinking.
- Thermogravimetric Analysis: TGA analysis was carried out using a Shimadzu DTG-60H (Shimadzu Corporation, Kyoto, Japan) thermogravimetric analyzer to evaluate the thermal stability and degradation behavior of cellulose, CMC, and the synthesized hydrogel. TGA analysis was performed according to Altuntas et al. [19] and Salleh et al. [4] The samples were heated under a nitrogen atmosphere at a constant heating rate of 10 °C/min from 30 to 600 °C, and the resulting weight-loss profiles were analyzed to determine the thermal degradation characteristics and stability of the materials.
- Swelling Measurements: The hydrogel swelling behavior was investigated using a gravimetric method and all measurements were performed in triplicate to ensure reproducibility; the results are reported as mean ± standard deviation. Swelling and deswelling protocols were adapted from Alam et al. [17] and Etminani-Esfahani et al. [20]. A known mass of dry hydrogel (Wd), 0.3271 g, was immersed in an excess of distilled water at room temperature for 12 days. At predetermined time intervals, the swollen sample was removed, gently blotted with filter paper to remove surface water, and weighed (Wt). The swelling ratio (SR) at each time point was calculated using the Equation (1). Measurements were continued until equilibrium was reached (constant weight, Wt), and the equilibrium swelling ratio was determined as:
  (1) $$\text{SR }\left(\%\right)=\frac{W_{t}-W_{d}}{W_{d}}\times 100$$
  where W_t_ is the weight of the swollen hydrogel at equilibrium (g) and W_d_ is the weight of the dry hydrogel (g).
- Deswelling Measurements: Water-retention behavior was evaluated by gravimetric deswelling measurements performed in triplicate. Fully swollen hydrogel samples were removed from distilled water, gently blotted to remove excess surface water, and their initial hydrated masses (W0) were recorded. The samples were then placed in Petri dishes under ambient laboratory conditions (25 °C and approximately 40% relative humidity). Sample mass (Wt) was recorded at predetermined time intervals, and the percentage mass loss was calculated according to Equation (2). Measurements were continued until the samples approached a constant mass, with the complete deswelling/drying period extending to approximately 48 h under the experimental conditions.
  (2) $$\text{Water Loss }\left(\%\right)=\frac{W_{0}-W_{t}}{W_{0}}\times 100$$

The deswelling test was continued until a significant fraction of the absorbed water had evaporated.

### 2.6. Soil Burial Biodegradation Test

The biodegradation of the synthesized hydrogel in soil was assessed using a soil burial test following established protocols [20]. Soil samples were collected from Harran University Osmanbey campus and passed through a 200-mesh sieve before use. The campus soil is a heavy clay soil with slightly alkaline to alkaline pH and low organic matter content, typical of Harran Plain soils [21,22]. Two different sets of semi-permeable carriers were prepared to compare the effect of the burial container on the degradation rate: (i) perforated polyethylene bags with holes punched in them, and (ii) muslin/cheesecloth bags. Three parallel replicates (*n* = 3) were prepared for each set. Hydrogel samples with an initial dry weight of approximately 0.50 g were placed in each carrier and buried at a depth of approximately 10 cm below the soil surface in plastic pots containing agricultural soil. Throughout the 28-day experimental period, soil moisture was maintained at 30% water-holding capacity by regular watering, and the pots were kept at room temperature (25 ± 2 °C).

At weekly intervals (days 7, 14, 21, and 28), three samples from each set were carefully removed from the soil and gently washed with distilled water to remove any soil particles adhering to their surfaces. In the perforated plastic bag experimental set, after removing and cleaning the samples, the hydrogel was carefully transferred to a Petri dish; the remaining particles were then diluted with water and transferred to the same dish. They were then dried in an oven at 50 °C until a constant weight was reached (2 days) and weighed on a precision balance (±0.0001 g). In the muslin cloth experimental set, the hydrogel-containing bag was gently rinsed with distilled water, then dried together with the hydrogel and weighed. At the end of the experiment, the muslin bag was thoroughly cleaned to remove all hydrogel residues, dried until it reached a constant weight, and weighed separately to determine the final mass of the carrier. The biodegradation rate (percentage of degradation) was calculated separately for each replicate using Equation (3), and the results are expressed as the mean ± standard deviation (SD):

(3) $$\text{Degradation rate }\left(\%\right)=\frac{W_{i}-W_{f}}{W_{i}}\times 100$$

where W_i_ represents the initial dry weight of the hydrogel, and W_f_ represents the dry weight of the sample extracted from the soil and dried at the relevant time point.

### 2.7. Data Analysis

Experimental data obtained from the swelling, deswelling, and soil-burial biodegradation tests were analyzed using three independent replicates (*n* = 3), and the results are expressed as mean ± standard deviation (SD). Swelling ratios, water-loss percentages, and apparent biodegradation percentages were calculated using the equations described in the corresponding experimental sections. For the soil-burial test, the mean remaining dry mass and apparent biodegradation percentage were determined for each sampling time and carrier system. XRD and TGA parameters were obtained from the corresponding instrumental data and processed as described in Section 2.5.

## 3. Results and Discussion

### 3.1. Synthesis and Chemical Cross-Linking of CMC/ECH Hydrogel

The main objective of this study was to develop a functional water-retention hydrogel from olive pomace through an integrated three-step valorization pathway. Unlike previous studies that relied on commercially available cellulose or CMC as starting materials [6,8,9,12], our approach utilizes de-oiled olive pomace as the cellulose precursor, addressing both waste management challenges and providing a value-added utilization route for this agro-industrial residue.

**Figure 2:**
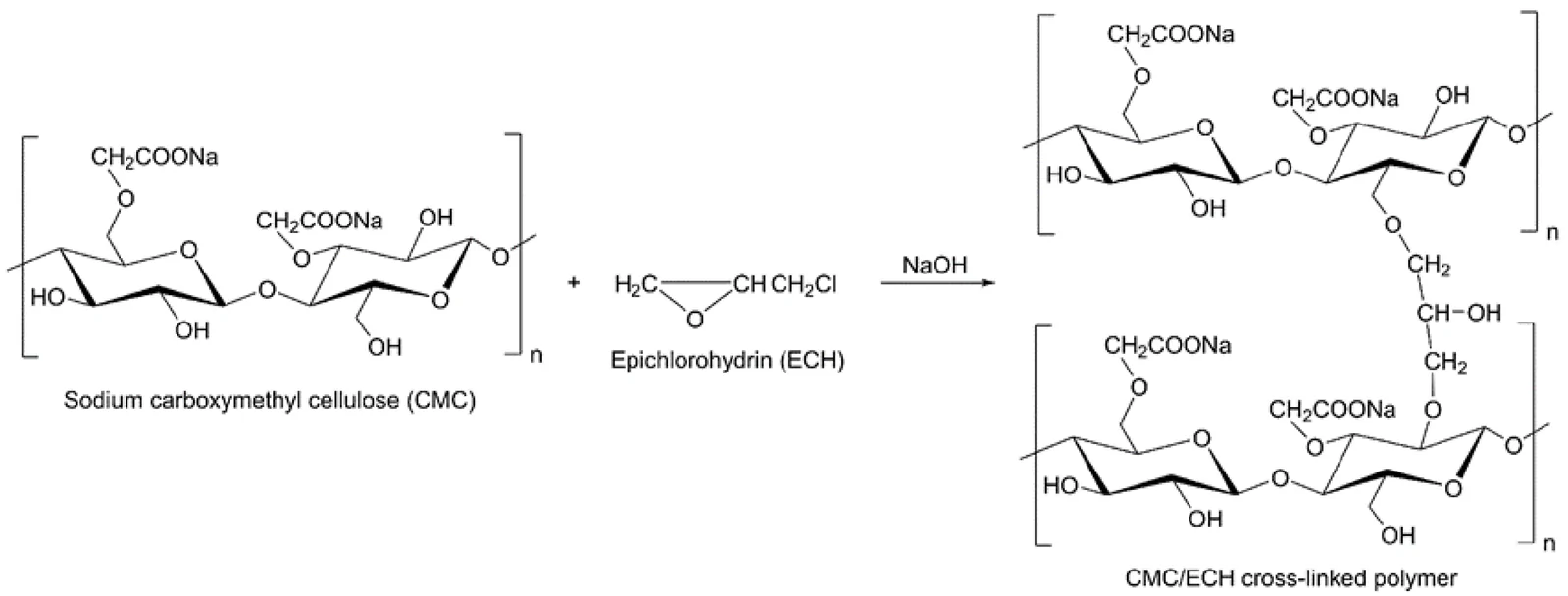
Schematic representation of CMC/ECH hydrogel formation via cross-linking reaction.

The crosslinking reaction between CMC and ECH proceeds through a base-catalyzed etherification mechanism. Under alkaline conditions (NaOH), the hydroxyl groups (-OH) on the CMC chains are deprotonated to form highly nucleophilic alkoxide ions (-O^−^). These alkoxide ions attack the electrophilic carbon of the epoxide ring in ECH, leading to ring opening and the formation of a new C–O–C ether bond. The chloride substituent on ECH is subsequently displaced, generating a new epoxide functionality that can react with another alkoxide ion from a neighboring CMC chain. This sequential ring-opening and chloride-displacement mechanism results in the formation of stable covalent crosslinks between adjacent CMC polymer chains, thereby creating an insoluble three-dimensional network. Figure 2 illustrates the proposed crosslinking mechanism.

The cellulose extraction yield obtained from de-oiled olive pomace in the present study was 9.16%. This value should be interpreted in light of the considerable variability reported for olive-derived lignocellulosic residues, which is influenced by biomass origin, olive processing conditions, initial composition, and the severity and number of extraction and purification steps. Previous studies have reported widely varying cellulose contents in olive pomace [23]. A multistep alkali-treatment and bleaching process specifically applied to olive pomace resulted in an overall cellulose extraction yield of approximately 20.36%. Other studies have reported cellulose contents of approximately 9.7–16.2% in exhausted or untreated olive pomace [24]. Therefore, the 9.16% recovery obtained in the present study is consistent with the relatively low cellulose fraction that may occur in olive pomace, although direct quantitative comparison should be made cautiously because cellulose content and cellulose extraction yield represent different parameters and depend strongly on the processing methodology.

Under the experimental conditions described in Section 2.4, rapid gelation was observed, accompanied by a marked increase in viscosity, indicating the formation of a three-dimensional polymer network. The dried hydrogel exhibited a yield of 32.55%. The structural and physicochemical characteristics of the resulting CMC/ECH hydrogel were subsequently evaluated by FTIR, XRD, SEM, TGA, and swelling analyses. The gelation process was accompanied by a rapid increase in viscosity, and complete gel formation was observed within approximately 10 min of heating. After synthesis, the hydrogel was thoroughly washed with ethanol/water mixtures, followed by distilled water, to remove residual ECH, NaOH, and soluble by-products, thereby ensuring the elimination of unreacted crosslinker. The dried hydrogel yielded 32.55%. The formation and structural characteristics of the crosslinked hydrogel were subsequently evaluated using FTIR, XRD, SEM, TGA, and swelling analyses, confirming the successful formation of the cross-linked network under the conditions applied here.

### 3.2. FTIR Analysis

**Figure 3:**
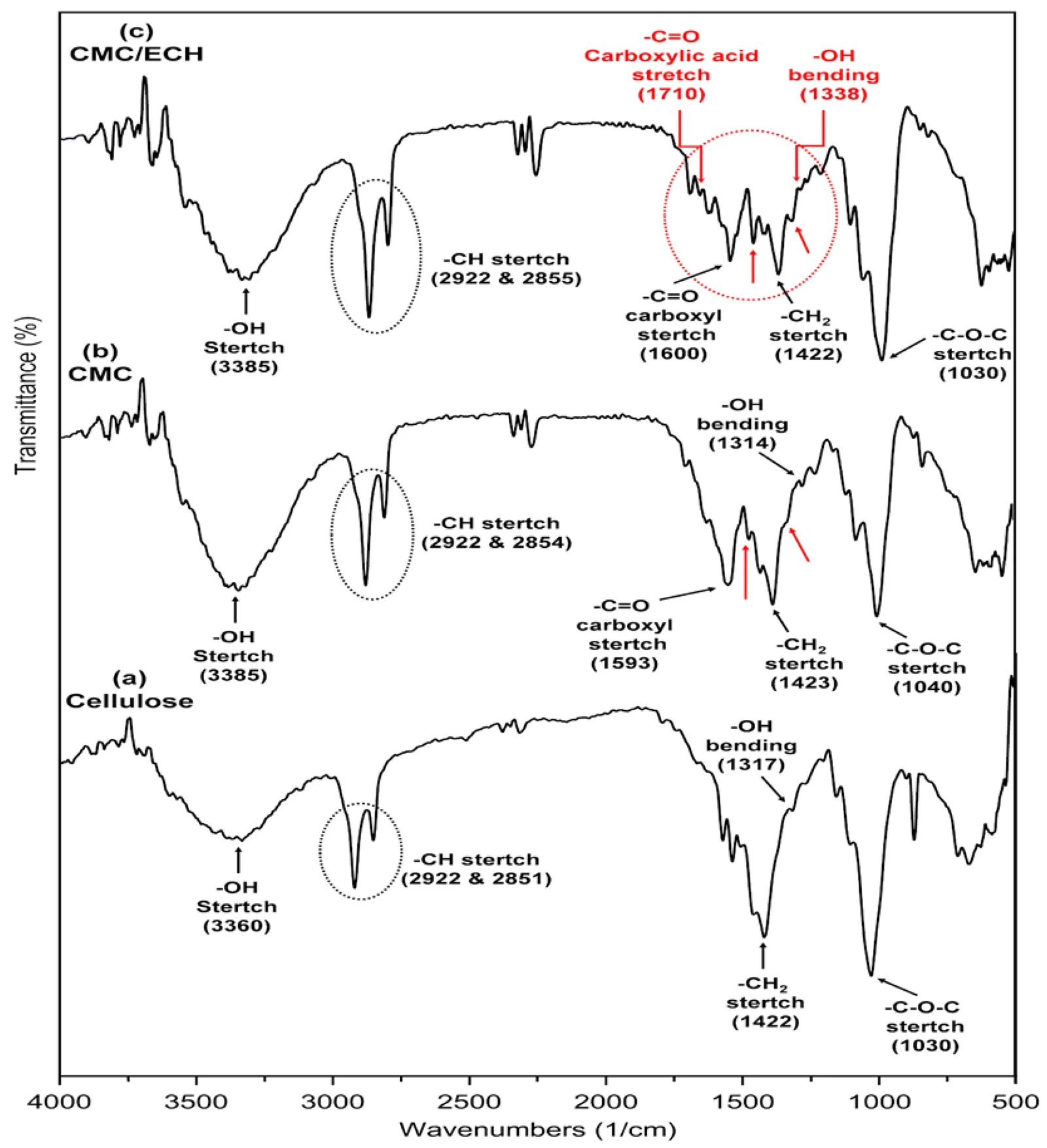
FTIR spectra of cellulose, CMC, and CMC/ECH crosslinked hydrogel.

FTIR spectroscopy was employed to monitor structural transformations during the stepwise conversion of raw olive pomace cellulose into carboxymethyl cellulose (CMC) and subsequent crosslinking to a three-dimensional hydrogel network. FTIR spectra of raw cellulose, synthesized CMC, and the final CMC/ECH hydrogel were compared to confirm successful chemical modifications (Figure 3).

Native cellulose exhibits characteristic bands at 3360 cm^−1^ (O–H stretching), 2922 and 2851 cm^−1^ (C–H stretching), 1422 cm^−1^ (CH_2_ scissoring), 1317 cm^−1^ (O–H bending), 1030 cm^−1^ (C–O–C stretching), and ~870 cm^−1^ associated with skeletal vibrations of the β-glycosidic polysaccharide backbone. After carboxymethylation, a new absorption band appeared at 1593 cm^−1^, which is assigned to the asymmetric stretching vibration of carboxylate (-COO^−^) groups, confirming successful conversion to sodium carboxymethyl cellulose. Minor shifts at 1317 → 1314 cm^−1^ and 1030 → 1040 cm^−1^ suggest changes in the hydrogen-bonding environment due to hydroxyl-group substitution. The persistence of the ~870 cm^−1^ band indicates that the β–glycosidic backbone remains intact after etherification.

The FTIR spectrum of the CMC/ECH hydrogel is broadly similar to that of CMC, indicating that the main polymer structure is preserved after crosslinking. However, the appearance or intensification of bands around 1710 and 1338 cm^−1^ may reflect changes in the environment of carboxyl and hydroxyl groups, consistent with crosslinking and network formation. These bands are often associated with C=O stretching and O–H bending, but in sodium CMC, they may also arise from altered hydrogen bonding or partial protonation under reaction conditions. Similar spectral changes have been reported in the literature for ECH-crosslinked cellulose-based hydrogels [3,5].

### 3.3. Crystalline Phase Assessment (XRD)

XRD analysis was performed to compare the crystalline structural characteristics of Cellulose, CMC, and Hydrogel samples synthesized from cellulose. The crystallite sizes were calculated using the full width at half maximum (FWHM) values obtained from the diffraction peaks, while the RDoCM (Relative Distribution of Crystalline Layer Motifs) values were determined to describe the relative crystalline layer motifs [25]. This approach enabled the evaluation of not only the crystallinity characteristics of the samples but also the nanoscale structural organization of their crystalline phases.

In the CMC sample, a single dominant crystalline layer motif was identified, with an RDoCM value of 100%. However, the high amorphous phase fraction of 73.03% and the relatively small crystallite size of only 1.63 nm indicate that CMC possesses a predominantly disordered structure with a low degree of crystallinity. During carboxymethylation, disruption of hydrogen bonding between cellulose chains and rearrangement of the macromolecular structure may have reduced crystalline regions and increased amorphous character.

In the cellulose sample, three distinct crystalline layer motifs were identified. The second layer showed the highest RDoCM value (55.07%), followed by the first (38.03%) and third layers (6.91%). The crystallite sizes calculated for the second and third layers (2.18 and 29.91 nm, respectively) indicate crystalline domains with different dimensions and degrees of structural organization within the cellulose structure. Nevertheless, the amorphous phase contributions of 34.04% and 49.29% associated with these layers show that the cellulose sample is not completely crystalline but rather a heterogeneous organization of both crystalline and disordered regions, as summarized in Table 2.

**Figure 4:**
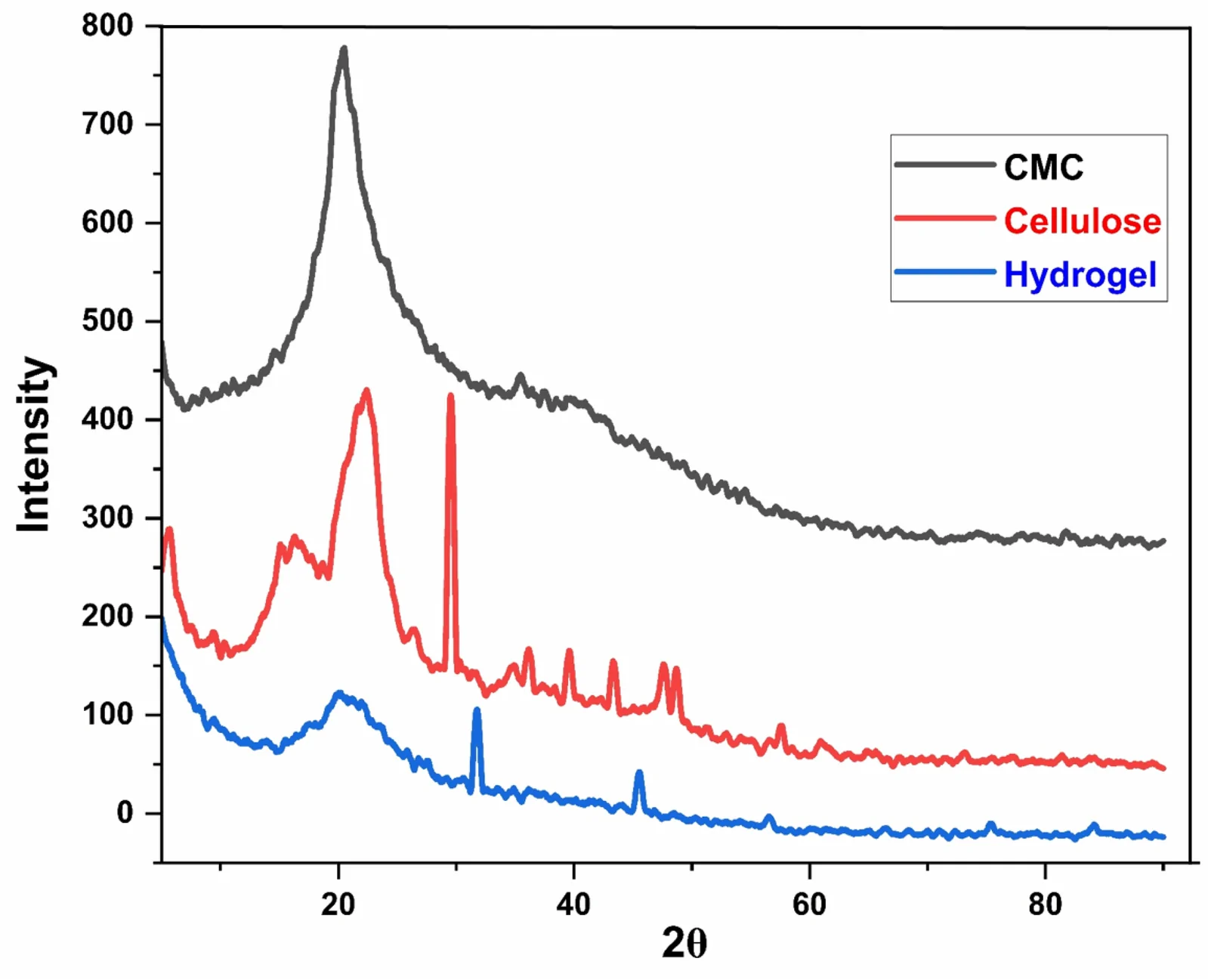
X-ray diffraction (XRD) patterns of Cellulose, Hydrogel, and CMC samples.

The Hydrogel sample showed a substantial change in crystalline structural distribution. The first layer accounted for 93.70% of the total crystalline motifs, whereas the second layer contributed only 6.31%. Despite its relatively low contribution, the second layer retained a larger crystalline domain with a size of approximately 30.10 nm. In contrast, the amorphous phase fraction increased to 80.93%, while the crystallite size associated with the dominant layer decreased to 1.16 nm. These results are clearly illustrated in the XRD patterns shown in Figure 4. These results indicate that hydrogel synthesis substantially disrupted the regular crystalline packing of the cellulose chains.

The increase in amorphous-phase contribution from 34.04–49.29% in the cellulose sample to 80.93% in the hydrogel sample indicates pronounced molecular and supramolecular structural reorganization during hydrogel formation. The intermolecular and intramolecular interactions during hydrogel synthesis, along with the development of a three-dimensional polymeric network, likely restricted the regular packing of cellulose chains and promoted the transformation of some crystalline domains into more disordered structures. This increase in amorphous regions is particularly relevant to hydrogel structure, as a more disordered polymeric network generally facilitates water penetration and enhances polymer chain mobility. Furthermore, the higher amorphous phase fraction observed in the Hydrogel sample (80.93%) compared with that of CMC (73.03%) indicates that the applied reaction conditions induced a further disruption of the crystalline organization of the cellulosic structure. This result supports the occurrence of substantial structural reorganization during hydrogel synthesis and demonstrates that the crystalline arrangement of the starting cellulosic material was effectively transformed during the formation of the hydrogel network.

### 3.4. Morphological Analysis (SEM)

**Figure 5:**
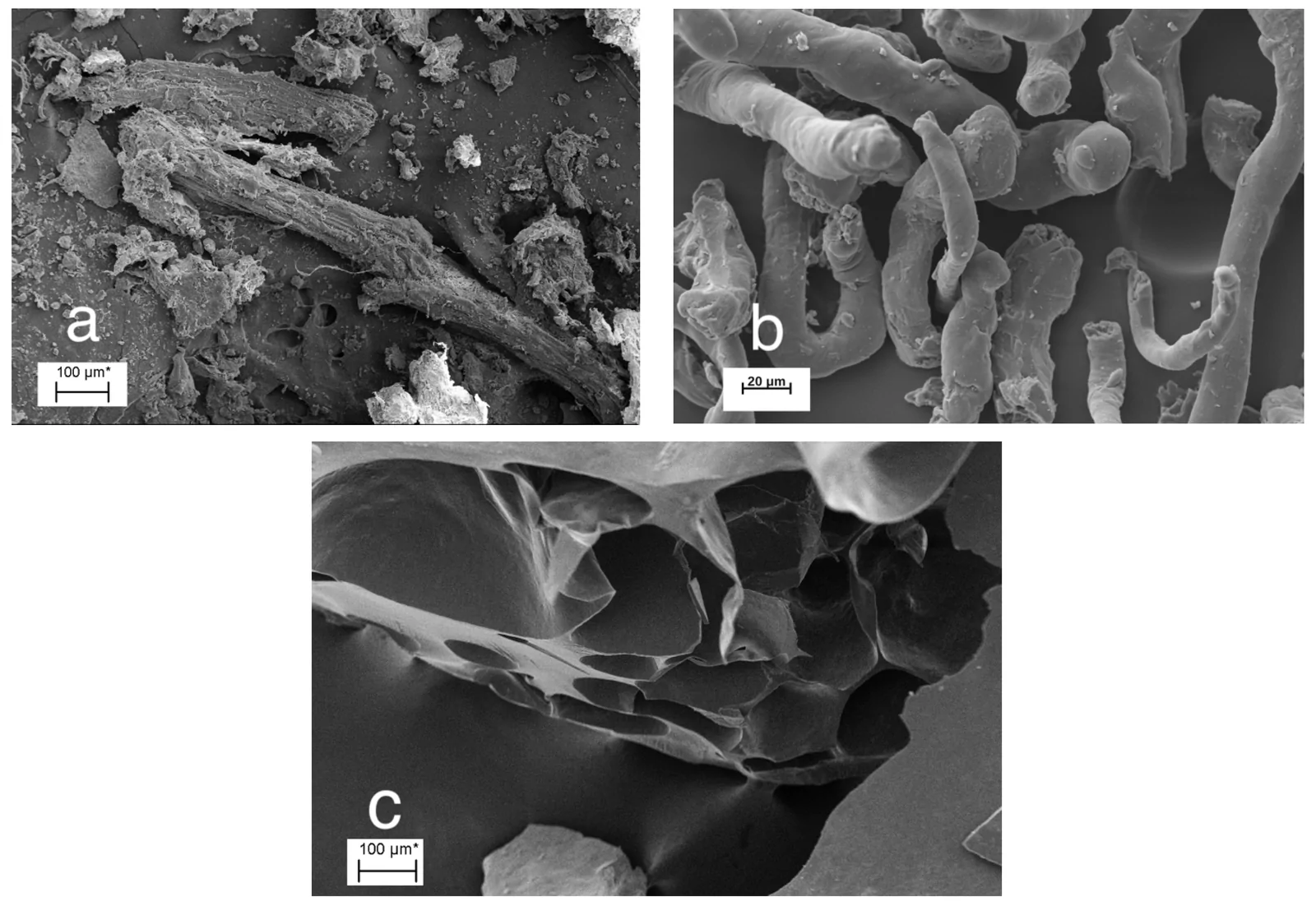
SEM micrographs of (**a**) extracted olive pomace cellulose, (**b**) synthesized carboxymethyl cellulose (CMC), and (**c**) CMC/ECH hydrogel. ((**a**): 100 µm; (**b**): 20 µm; (**c**): 100 µm.) * denotes the unit micron (µm) used in the scale bars.

Scanning electron microscopy (SEM) was employed to investigate the microstructural features of the extracted cellulose, synthesized carboxymethyl cellulose (CMC), and the final ECH-crosslinked hydrogel, providing critical insights into the morphological transformations occurring at each stage of the synthesis process. Figure 5 presents the SEM micrographs.

The SEM image of the extracted cellulose (Figure 5a) reveals a highly oriented, compact, and fibrous morphology characteristic of native lignocellulosic materials. The fibers exhibit a relatively smooth surface with a dense, bundled structure, reflecting the organized arrangement of cellulose microfibrils within the plant cell wall. This compact fibrous architecture is typical of cellulose isolated from agricultural biomass after alkaline delignification, as removing lignin and hemicellulose exposes the underlying cellulose microfibrillar network while preserving its inherent structural integrity. The densely packed fiber bundles observed in the micrograph are consistent with the relatively high crystalline content (amorphous phase fractions ranging from 34.04% to 49.29% for different crystalline layers) determined by XRD analysis, as the ordered arrangement of cellulose chains within the crystalline domains contributes to the material’s rigidity and compact morphology.

After carboxymethylation, the SEM image of the synthesized CMC (Figure 5b) shows a marked morphological transformation compared with native cellulose. The originally compact and well-defined fibrous structure becomes significantly more swollen, distorted, and irregular, with individual fibers appearing less distinct and more aggregated. This morphological change is attributed to the introduction of carboxymethyl groups (-CH_2_COONa) onto the cellulose backbone, which disrupts the inter- and intramolecular hydrogen bonding network that maintains the native fibrous architecture. The bulky, negatively charged carboxymethyl substituents generate electrostatic repulsion between adjacent chains, forcing them apart and causing the fibers to swell and lose their original ordered morphology. Additionally, CMC’s increased hydrophilicity promotes water absorption during sample preparation, further contributing to the swollen, distorted appearance observed in the micrograph. This morphological transformation is consistent with the substantial increase in amorphous phase fraction (from 34.04–49.29% to 73.03%) observed in the XRD analysis, supporting the conclusion that carboxymethylation disrupts the ordered crystalline domains of the native cellulose.

The SEM image of the ECH-crosslinked CMC hydrogel (Figure 5c) shows a dramatically different morphology from both native cellulose and uncrosslinked CMC. The hydrogel displays a highly porous, interconnected three-dimensional network structure with a honeycomb-like architecture. The cross-section reveals uniformly distributed pores with relatively smooth pore walls, characteristic of chemically crosslinked polysaccharide hydrogels. The formation of this porous network is a direct consequence of the ECH-mediated crosslinking reaction, which covalently bonds adjacent CMC chains through stable ether (C–O–C) linkages, creating a permanent three-dimensional network that resists dissolution [3,5]. The porous architecture observed in the hydrogel micrograph can be attributed to several interrelated factors. First, the electrostatic repulsion between the ionized carboxylate groups (-COO^−^) on the CMC backbone forces the polymer chains apart during swelling, generating voids within the network. Second, the crosslinking reaction introduces covalent bonds between chains, stabilizing the expanded network and preventing its collapse upon drying [19]. Overall, these morphological features indicate the formation of a three-dimensional network that may facilitate water penetration and retention within the hydrogel. A numerical pore-size distribution was not determined from SEM images alone.

### 3.5. Thermogravimetric Analysis (TGA)

**Figure 6:**
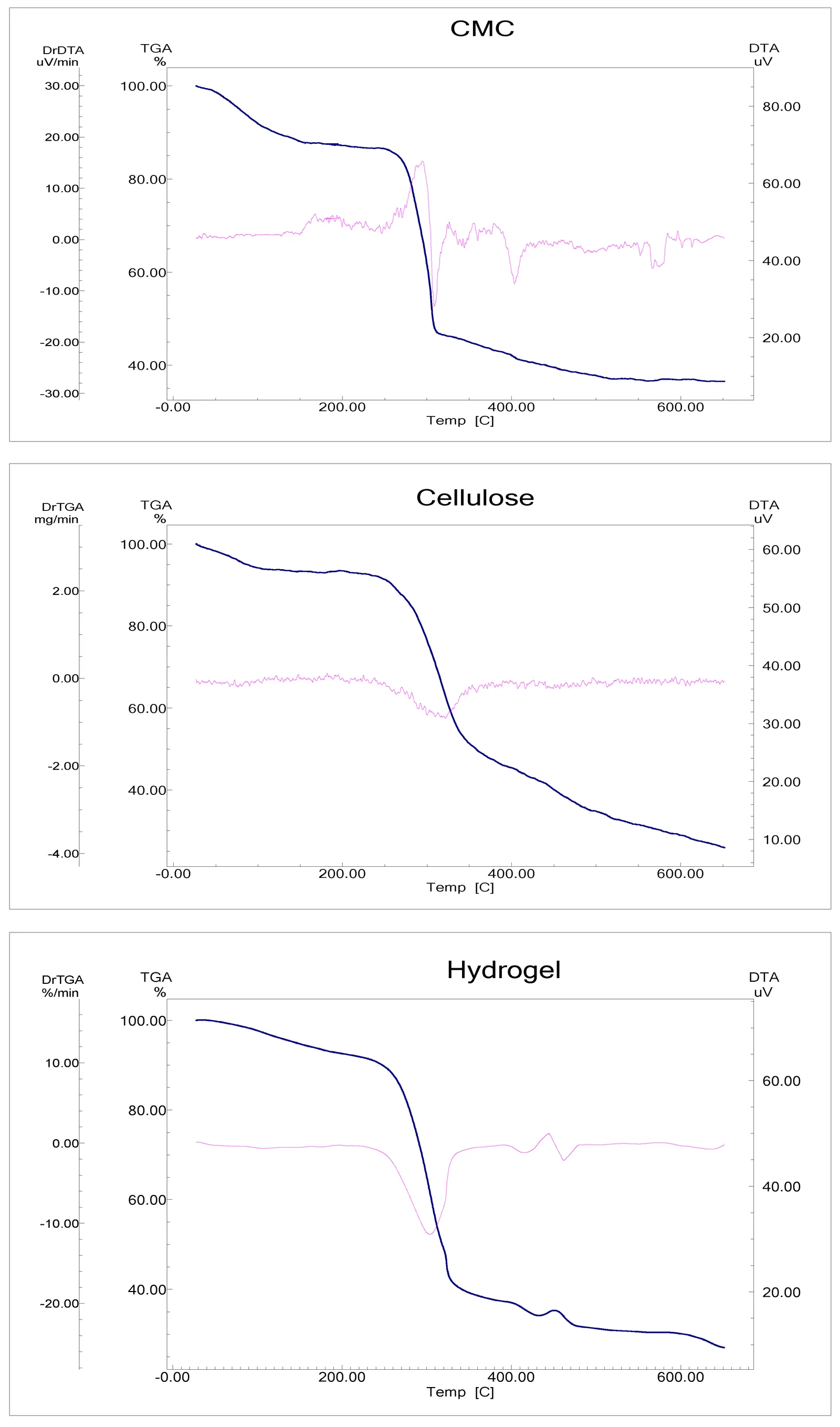
Comparative TGA (black line) and DTA (magenta line) profiles of cellulose, hydrogel, and CMC samples illustrating their thermal degradation behavior and thermal stability characteristics. Thermal analysis data were acquired and processed using TA60 software (Version 2.01).

Thermogravimetric analysis was performed to comparatively evaluate the thermal stability and the degradation behavior of cellulose, CMC, and CMC/ECH hydrogel. The results are presented in Table 3 and Figure 6, where the onset degradation temperature (ID), end degradation temperature (EDT), derivative thermogravimetric parameters, total weight loss (TW), degradation temperature range (TsD), derivative weight loss (DWL), moisture and bound-water losses, and degradation durations were evaluated to elucidate the effects of structural transformations on the thermal behavior of the samples [18].

The onset degradation temperatures followed the order CMC (247.23 °C) > Cellulose (243.67 °C) > Hydrogel (236.05 °C). The lower ID value observed for the hydrogel suggests that the three-dimensional network structure formed during hydrogel synthesis, together with the increased proportion of amorphous domains, facilitated the initiation of thermal degradation. This interpretation is further supported by the high amorphous phase fraction (80.93%) determined by XRD analysis. In contrast, the higher onset degradation temperature of CMC indicates that carboxymethylation imparted a certain degree of resistance to the initial stages of thermal degradation.

In addition, the samples showed more pronounced differences in overall degradation behavior. The EDT values were determined as 379.55 °C for cellulose, 336.00 °C for hydrogel, and 315.64 °C for CMC. Similarly, the total degradation temperature range (TsD) was calculated as 135.88 °C for Cellulose, 99.95 °C for Hydrogel, and 68.41 °C for CMC. The corresponding total degradation durations (tD) were 13.17, 9.27, and 6.82 min, respectively. These results indicate that cellulose undergoes thermal degradation over a substantially broader temperature and time interval, demonstrating its superior overall thermal stability. This behavior can be attributed to the more ordered crystalline domains and stronger intermolecular hydrogen-bonding interactions observed in the Cellulose structure, consistent with the XRD results.

Although thermal degradation of the hydrogel commenced at a lower temperature, the Hydrogel exhibited higher EDT, TsD, and tD values than CMC. This finding suggests that, despite the partial disruption of the crystalline structure during hydrogel synthesis, the resulting three-dimensional network retained a certain degree of structural and thermal integrity. Notably, the hydrogel exhibited the highest DWL value (50.86%), indicating substantial mass loss during the main degradation stage. However, the broader degradation interval compared with CMC suggests that this mass loss occurred more gradually and in a more controlled manner, reflecting the contribution of the three-dimensional polymer network to the overall degradation process.

The water-loss parameters revealed significant structural differences among the samples. Moisture-related weight loss (ΔHWL) was considerably lower for the hydrogel (2.67%) than for cellulose (5.81%) and CMC (8.59%). In contrast, the bound-water content (ΔbwWL) increased from 6.77% in cellulose to 8.91% in the hydrogel. This increase indicates that the crosslinked network formed during hydrogel synthesis promoted stronger interactions between water molecules and the polymer matrix. In other words, while the amount of free or physically adsorbed water decreased in the room-temperature-dried hydrogel, the fraction of water more strongly associated with the polymer network increased. This behavior indicates not only the material’s water-retention capacity but also the successful development of the hydrogel network.

In particular, the simultaneous decrease in ΔHWL and increase in ΔbwWL from 6.77% in Cellulose to 8.91% in the hydrogel suggest that water was retained in a more strongly associated and thermally stable state following hydrogel formation. Together with the high amorphous-phase fraction determined by XRD, these findings support the structural reorganization of cellulose chains into a predominantly amorphous, three-dimensional hydrogel network with strong intermolecular and/or crosslinking interactions. If this structural transformation had not occurred, we would expect a higher proportion of free moisture and a lower fraction of strongly associated water after drying.

Overall, the TGA results are consistent with the reduced crystallinity and increased amorphous content observed by XRD, confirming that the cellulose structure underwent substantial reorganization during hydrogel synthesis. Collectively, the increased bound-water content, reduced moisture loss, and enhanced amorphous character relative to cellulose indicate that the synthesized hydrogel developed strong water–polymer interactions and a successfully formed three-dimensional network. Although the increased amorphous character lowered the onset temperature of degradation, the extended degradation range and higher thermal stability relative to CMC show that the three-dimensional network helped maintain the hydrogel’s overall thermal integrity.

### 3.6. Swelling Properties of the Hydrogel

**Figure 7:**
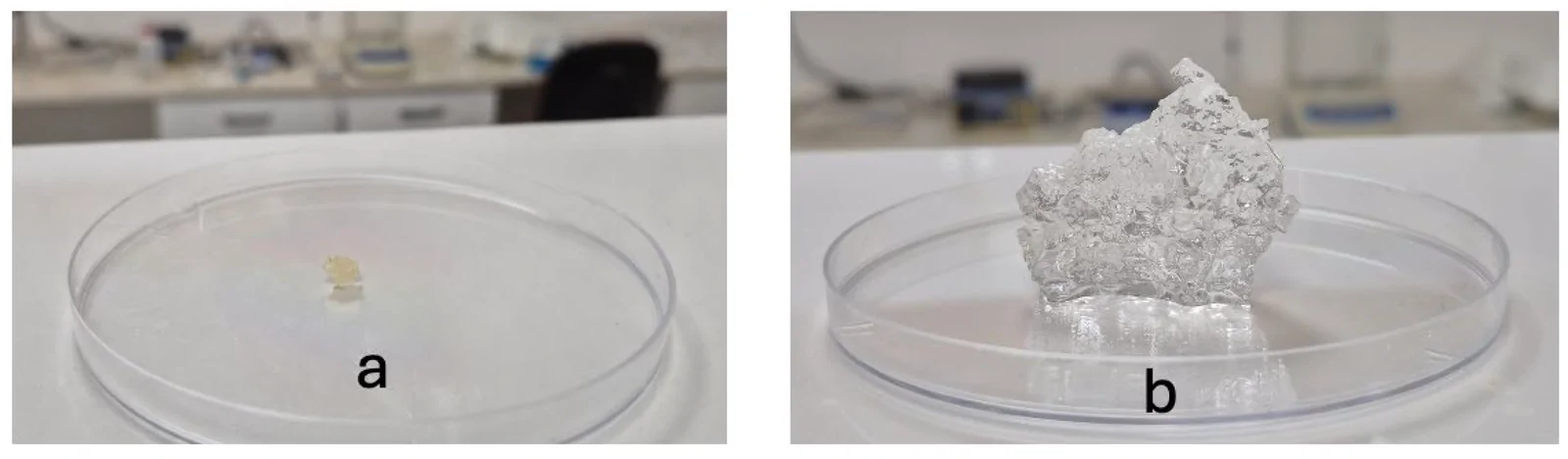
Photographic images of (**a**) dried CMC/ECH hydrogel and (**b**) fully swollen hydrogel after approximately 12 days of immersion (swelling ratio: 2616%).

After approximately 12 days of immersion, the hydrogel reached its maximum recorded swelling state with a wet mass of 8.8851 g, corresponding to a net water uptake of 8.5580 g. The equilibrium swelling ratio was calculated as 2616%, equivalent to 26.16 g of water per gram of dry hydrogel, as shown in Figure 7 and Table 4.

**Figure 8:**
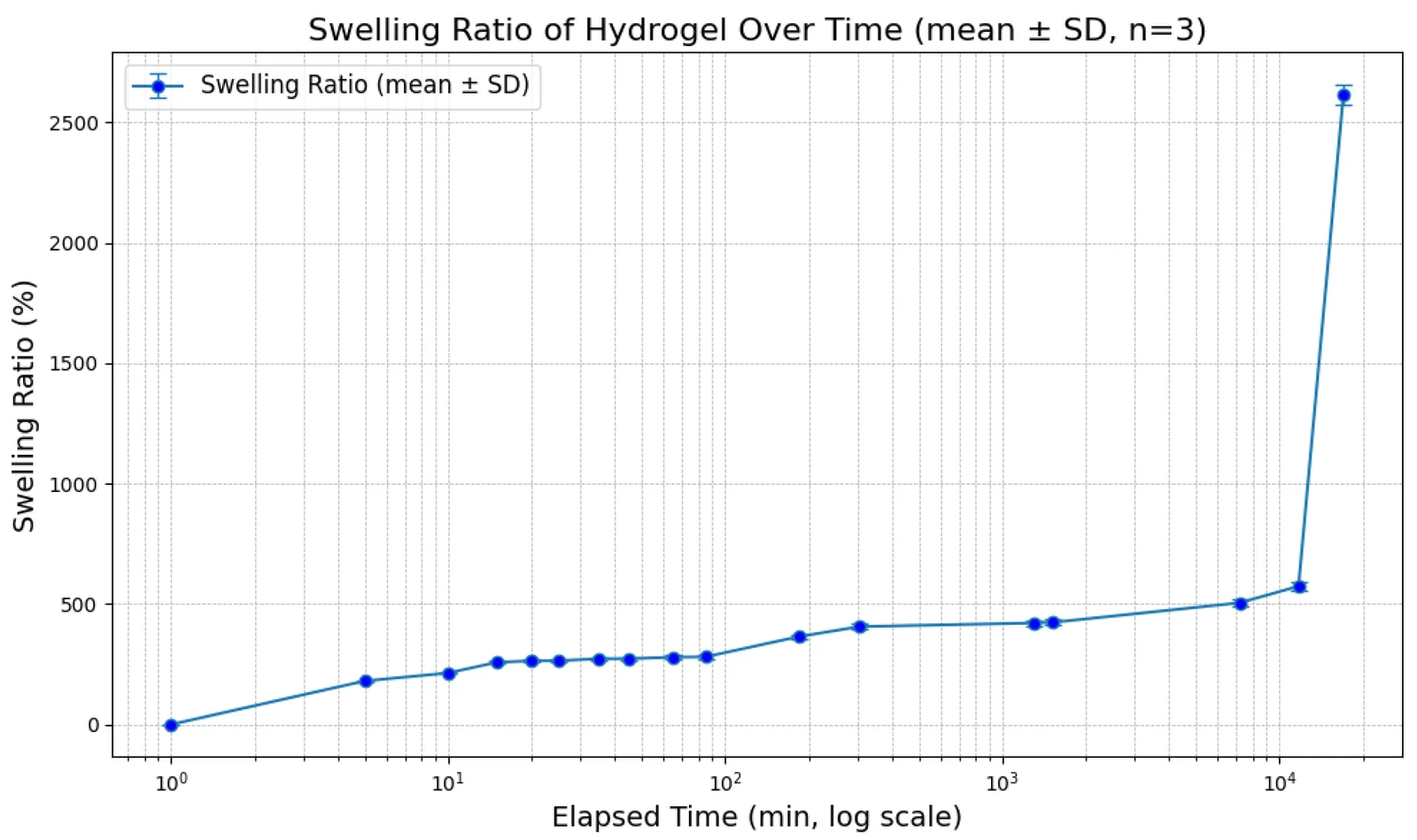
Swelling profile of hydrogel over time.

The swelling behavior exhibited (Figure 8) distinct time-dependent stages. The material showed rapid initial water uptake during the first 45 min, followed by a much slower swelling stage during prolonged immersion. The swelling ratio then increased from 574.3% at 11,726 min (approximately 8 days) to a maximum of 2616% at 17,026 min (approximately 12 days), indicating a pronounced late-stage swelling response.

This marked increase may reflect progressive hydration and relaxation of the crosslinked CMC/ECH network during prolonged immersion. As water progressively penetrates the polymer network, previously less accessible hydrophilic domains may become available for further hydration, accompanied by increased chain mobility and network expansion. The predominantly amorphous character observed by XRD may also facilitate water penetration and molecular mobility during prolonged hydration. However, because no time-resolved structural measurements were performed during the swelling experiment, the precise mechanism responsible for this late-stage increase cannot be conclusively established. Therefore, we interpret this behavior cautiously as a possible delayed secondary swelling process rather than evidence of a specific structural transition.

**Figure 9:**
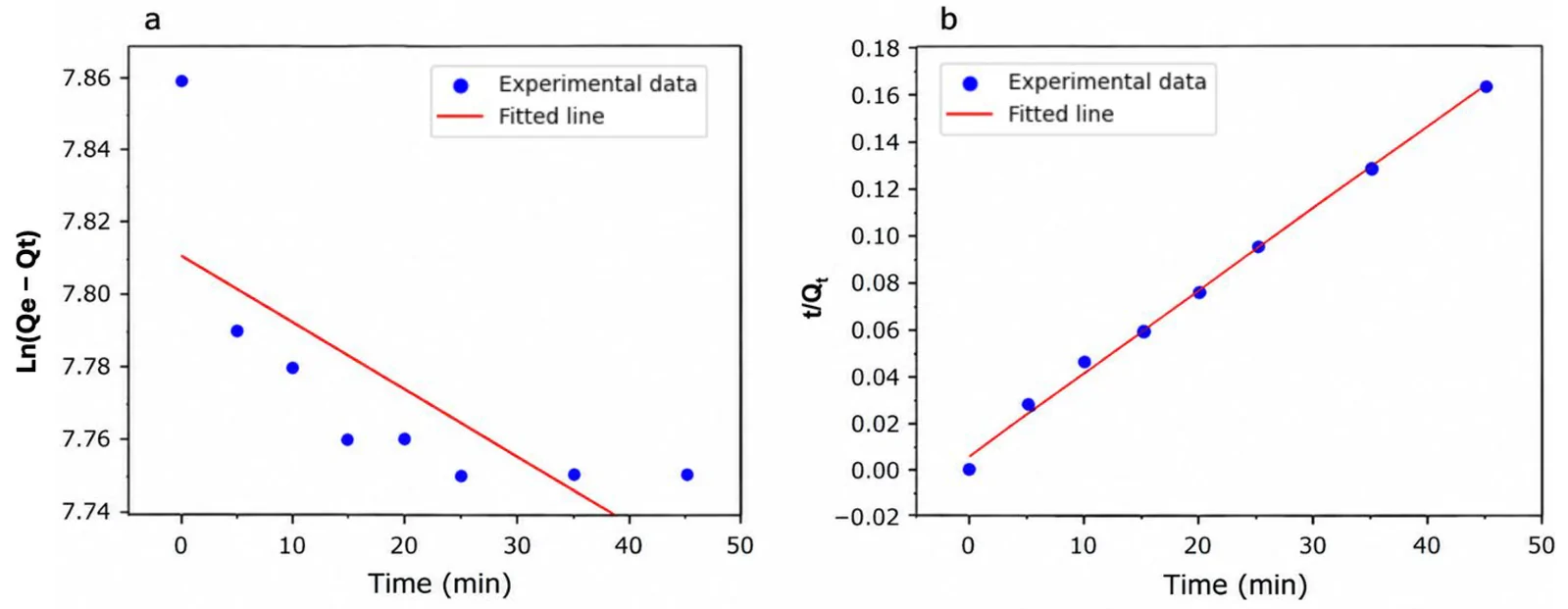
Kinetic modeling of the initial swelling behavior of the CMC/ECH hydrogel: (**a**) pseudo -first-order (PFO) model and (**b**) Schott pseudo-second-order (PSO) model.

The initial swelling kinetics of the CMC/ECH hydrogel were evaluated using the pseudo-first-order (PFO) and Schott pseudo-second-order (PSO) models (Figure 9 and Table 5). The PFO model showed a relatively poor fit to the experimental data, with a coefficient of determination (R^2^) of 0.5757, indicating that it does not adequately describe the hydrogel’s initial swelling behavior. In contrast, the Schott PSO model exhibited a substantially higher R^2^ value of 0.9963, demonstrating a much better mathematical description of the initial swelling stage. The calculated swelling capacity obtained from the Schott model (Qe, cal = 285.71%) was also close to the swelling values experimentally observed during the initial 45 min, which reached approximately 273.3%. These results indicate that the initial water uptake is more appropriately described by the Schott model and is consistent with a swelling process involving progressive hydration accompanied by relaxation and expansion of the crosslinked polymer network. However, the kinetic analysis was restricted to the initial swelling stage (0–45 min) and therefore does not describe the pronounced long-term secondary swelling observed between approximately 8 and 12 days.

### 3.7. Deswelling Properties of Hydrogel

**Figure 10:**
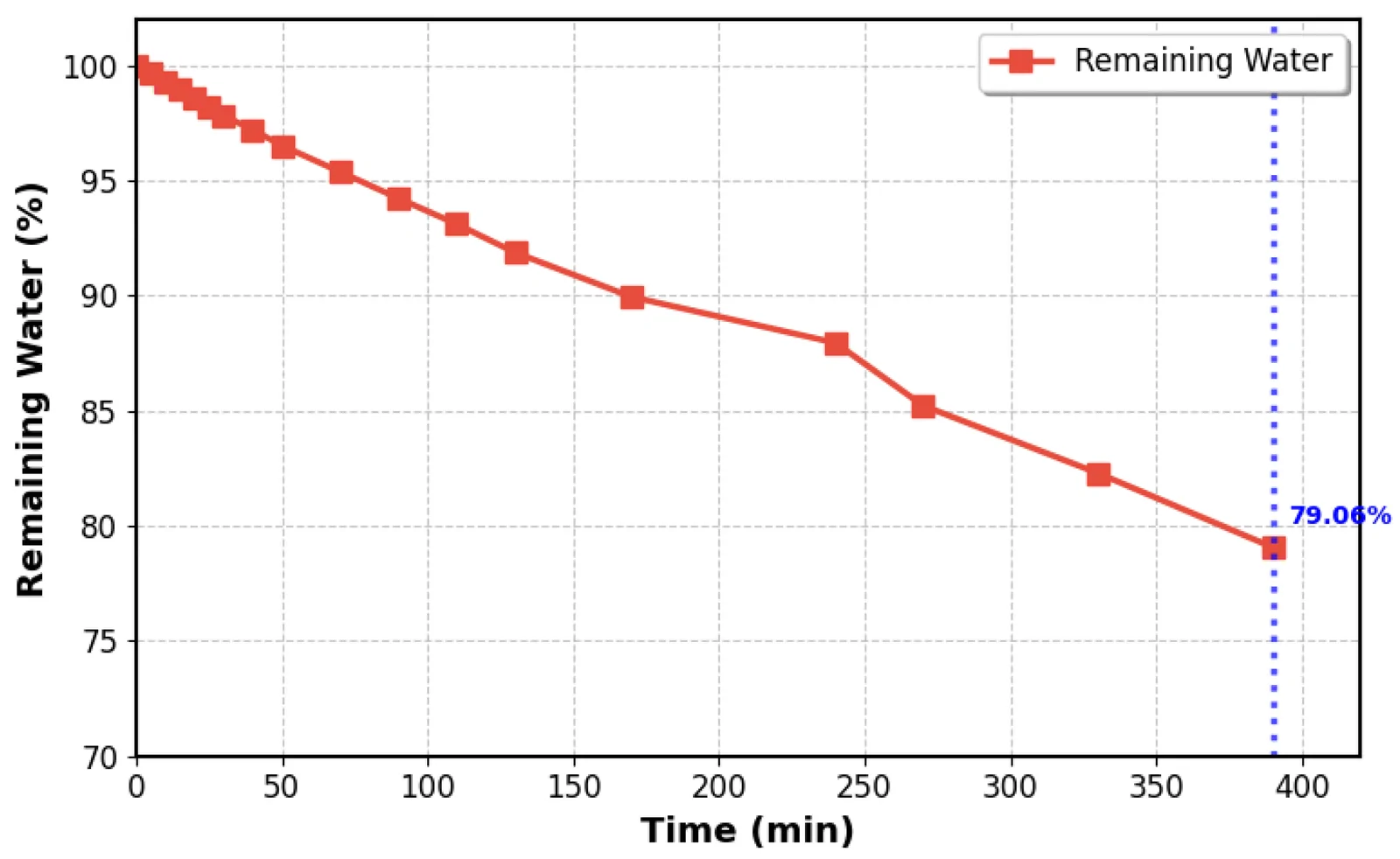
Deswelling and water-retention profile.

An effective hydrogel must not only absorb water efficiently but also retain it against environmental evaporation. The deswelling behavior of a fully hydrated hydrogel sample with an initial mass of 6.0390 g was evaluated by monitoring the sample mass at predetermined time intervals under ambient evaporation conditions. As summarized in Table 6 and Figure 10, the hydrogel exhibited strong water-retention behavior, with a cumulative mass loss of only 1.06% during the first 15 min and 5.77% after 90 min. By 4 h, cumulative mass loss was 12.05%, while after 6.5 h the hydrogel retained 4.7746 g, corresponding to a 20.93% reduction relative to its initial hydrated mass. Thus, 79.07% of the initial hydrated mass remained after 6.5 h. The water-retention assessment was based on sequential gravimetric measurements of the hydrated hydrogel over time. Accordingly, the reported 79.07% value represents the proportion of the initial hydrated sample mass remaining after 6.5 h rather than the proportion of absorbed water alone. The deswelling test continued until the sample approached a constant weight, with complete drying extending to approximately 48 h.

The final deswelling ratio calculated over extended drying was 94.5%. This controlled water-release profile indicates strong hydrogen-bonding interactions between water molecules and the carboxylate groups on the CMC backbone, lowering the chemical potential of the trapped water and reducing evaporation rates.

Our findings show that the bio-sourced hydrogel derived from valorized olive pomace possesses the precise intermolecular bond architecture required to hold water molecules securely under high environmental heat gradients. This performance characteristic makes our biopolymer exceptionally beneficial for environmental remediation, moisture control, and agricultural soil hydration, without the risk of sudden, early dehydration.

### 3.8. Soil-Burial Biodegradation of the Hydrogel

The soil-burial biodegradation of the synthesized hydrogel in soil was assessed over 28 days using two different semi-permeable carrier systems (perforated plastic bag and muslin/cheesecloth), with three parallel samples (*n* = 3) collected every 7 days. The choice of two carrier systems was motivated by the need to evaluate the effect of the burial container on the degradation rate and to assess the hydrogel’s potential across different agricultural application scenarios. In both experimental sets, a time-dependent reduction in hydrogel mass was observed, indicating progressive biodegradation. Etminani-Esfahani et al. [20] attributed this reduction to the penetration of water, microorganisms, and enzymes into the porous hydrogel structure, which hydrolytically and enzymatically broke the glycosidic and ether bonds. As shown in Table 7, the degradation rates at the final weighing were 53.88% for the muslin cloth and 41.25% for the perforated plastic bag.

**Figure 11:**
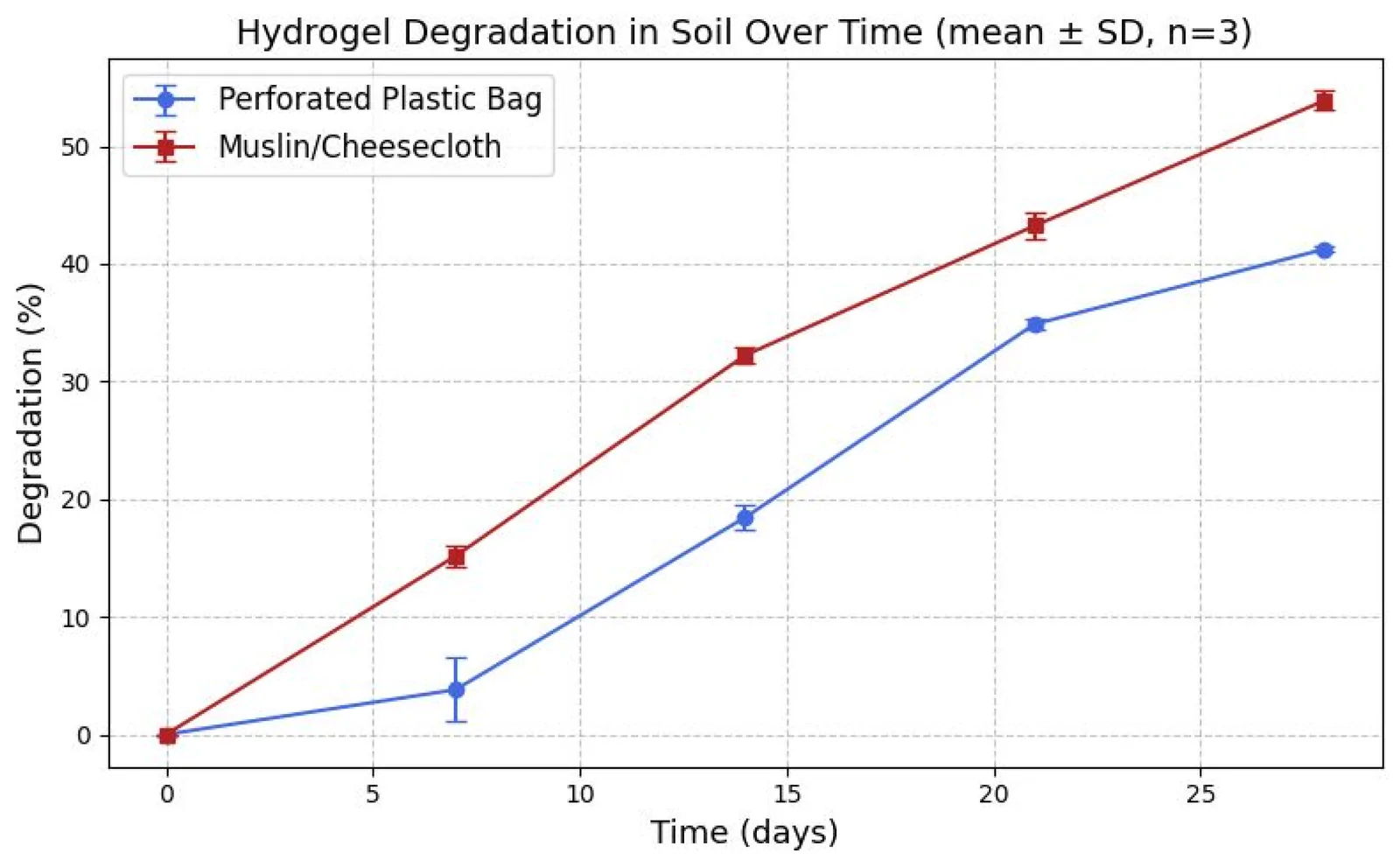
Rate of mass loss (%) of the hydrogel in soil over time (mean ± SD, *n* = 3).

The highest difference in biodegradation between the two types of carrier container was observed in the measurements taken during the first week: while mass loss in the muslin group reached 15.17% in the first week, this figure remained at just 3.81% in the perforated polyethylene bag group. This difference likely stems from the carrier container’s permeable structure. The open-pored structure of muslin fabric facilitates the access of soil moisture, microbial colonisation and enzymes to the sample, and increases the physical loss of fragmented gel during sample extraction and washing. This indicates that the measured biodegradation reflects not only actual biological degradation but also, to a certain extent, particle loss. In contrast, the more enclosed microenvironment of the perforated polyethylene bag significantly slowed biodegradation in the early stages by limiting access to water and microbes through the holes, while also retaining the fragmented material more effectively. As the gravimetric mass loss observed in the analysis cannot distinguish between biological degradation and physical particle loss, the values reported here should be interpreted as apparent mass loss observed under burial conditions, rather than as verified biological degradation rates. However, measurements taken over the final week showed cumulative mass loss rates of 41.25% and 53.88% in the plastic bag and muslin test groups, respectively, as presented in Figure 11. This indicates that, regardless of the carrier type, the microbial activity ultimately became the dominant factor. Although these results suggest that microbial activity increases over time, they are not sufficient to confirm the biological nature of the degradation.

The mass loss trends observed in this study are consistent with previous reports on cellulose-based hydrogels. The progressive biodegradation over the 28-day incubation period suggests that the hydrogel may be susceptible to microbial and enzymatic attack in soil environments. The high mass loss rate observed in the muslin cloth system (53.88% after 28 days) is consistent with the possibility that the hydrogel’s chemical structure, primarily composed of cellulose derivatives crosslinked with ether bonds, is biodegradable under natural soil conditions.

It has been reported that the degradation mechanism may involve the hydrolysis of the glycosidic bonds in the cellulose backbone by cellulase enzymes produced by soil microorganisms, as well as the cleavage of ether cross-links via enzymatic and/or hydrolytic pathways [20]; the identification of these specific degradation pathways may be elucidated in the future through detailed analyses such as FTIR and SEM.

The difference in mass loss rates between the two carrier systems provides potentially valuable insights for practical applications. For agricultural soil conditioning applications, where the hydrogel is intended to be mixed directly into the soil matrix, the high degradation rate observed in the muslin cloth system suggests that the material would be effectively biodegraded over a growing season, potentially leaving minimal persistent residues in the soil. Conversely, for controlled-release applications such as fertilizer coatings, the slower mass loss observed in the more enclosed environment may be advantageous, providing sustained release of encapsulated nutrients over an extended period.

Overall, the biodegradation results demonstrate that the olive pomace-derived hydrogel is not only a highly efficient water-retention material but also exhibits mass loss under the investigated soil-burial conditions. This end-of-life biodegradability, combined with sustainable sourcing from agricultural waste and excellent water absorption and retention properties, positions the hydrogel as a promising candidate for various agricultural and environmental applications.

## 4. Conclusions

This study demonstrates an integrated valorization route for transforming de-oiled olive pomace into a functional cellulose-based superabsorbent hydrogel through cellulose recovery, carboxymethylation, and ECH crosslinking. Despite the relatively modest cellulose recovery yield (9.16%), reflecting the heterogeneous lignocellulosic composition of olive pomace and the extraction conditions used, the recovered cellulose was successfully converted into CMC and subsequently into a three-dimensional hydrogel network. Structural and morphological analyses supported the successive modification of the cellulose-derived material, while the resulting hydrogel exhibited high water uptake, sustained water retention, and measurable degradation under soil-burial conditions. Overall, the findings demonstrate the feasibility of converting olive pomace into a value-added water-absorbing material while integrating agricultural-waste valorization with functional hydrogel production. Further studies should validate its performance under realistic soil–plant conditions and assess its long-term behavior and environmental impact.

## Figures and Tables

**Table 1:** Composition of olive pomace fractions on a dry weight basis.

Component	Gram	% *
Olive pomace	410.00	
Residual coarse kernel	320.21	78.10
Cellulose	37.56	9.16
Intermediate cellulose fiber	17.07	4.16
Loss	35.16	8.58

* Cellulose Yield (%) = (Recovered Cellulose Mass/Initial Dry Olive Pomace Mass) × 100; Intermediate Fiber Yield (%) = (Recovered Intermediate Fiber Mass/Initial Dry Olive Pomace Mass) × 100; Mass Loss (%) = [(Initial Dry Olive Pomace Mass − Total Recovered Mass)/Initial Dry Olive Pomace Mass] × 100.

**Table 2:** Crystalline layer distribution, nanoscale layer dimensions and amorphous content determined from XRD analysis of Cellulose, Hydrogel and CMC samples.

Sample Type	Layer(s)	FWHM	2θ	Nano Size of Layer	RDoCM *	CrI (%)	Amorphous (%)
CMC	1	5.177	20.340	1.631	100.000	26.97	73.028
Hydrogel	1	7.290	20.390	1.159	93.695	19.07	80.933
	2	0.287	31.740	30.101	6.305	94.56	5.446
Cellulose	1	5.570	16.320	1.508	38.025	65.96	34.040
	2	3.880	21.920	2.182	55.065	50.71	49.294
	3	0.287	29.600	29.907	6.910	93.81	6.186

* Relative distribution of crystalline layer motifs (%), calculated from the relative integrated peak areas of the fitted XRD reflections. FWHM, 2θ, crystallite size, and RDoCM values were obtained from single XRD measurements for each sample. 2θ: diffraction angle; FWHM: full width at half maximum; RDoCM: relative distribution of crystalline layer motifs; CrI: crystallinity index.

**Table 3:** Thermogravimetric parameters of Cellulose, Hydrogel, and CMC samples *.

Thermogravimetric Parameters	CMC	Hydrogel	Cellulose
ID ^1^ (°C)	247.23	236.05	243.67
ED^T 2^ (°C)	315.64	336	379.55
Sp1DD^T 3^ (°C)	294.98	-	-
1Sp2DD^T 4^ (°C)	308.19	303	321.61
TW ^5^ (%)	63.26	72.954	73.69
TsD ^6^ (°C)	68.41	99.95	135.88
DWL ^7^ (%)	39.79	50.86	40.75
ΔHWL ^8^ (%)	8.59	2.67	5.81
ΔbwWL ^9^ (%)	13.47	8.91	6.77
ID^t 10^ (min)	21.44	21.13	20.72
Ed^t 11^ (min)	28.26	30.4	33.89
tD ^12^ (min)	6.82	9.27	13.17
1Sp2DD^t 13^ (min)	25.59	-	-
2Sp2DD^t 14^ (min)	27.25	26.76	28.85

* All thermogravimetric parameters were obtained from individual TGA thermograms of cellulose, CMC, and hydrogel samples. The reported values represent characteristic parameters derived from the corresponding thermal degradation curves and are not replicate measurements. Except for TsD and tD, all parameters were directly obtained from the TGA/DTG software analysis of the thermograms. ^1^ Initial degradation temperature; ^2^ End degradation temperature; ^3^ First set-point temperature of the first derivative degradation curve; ^4^ Second set-point temperature of the first derivative degradation curve; ^5^ Total weight loss; ^6^ Total degradation temperature range; ^7^ Derivative weight loss; ^8^ Humidity-related weight loss; ^9^ Bonded water-related weight loss; ^10^ Initial degradation time; ^11^ End degradation time; ^12^ Total degradation time; ^13^ First set-point time of the first derivative degradation curve; ^14^ Second set-point time of the first derivative degradation curve.

**Table 4:** Swelling Ratio of Hydrogel Over Time (mean ± SD, *n* = 3).

Geçen Süre (dk)	Swelling Ratio (%) (Mean ± SD)	Swelling Ratio (%) Trial		
		1	2	3
0	0.0 ± 0.0	0.0	0.0	0.0
5	180.8 ± 3.2	186.97	179.20	176.23
10	213.8 ± 4.5	207.38	222.47	211.55
15	258.3 ± 5.8	269.48	250.02	255.40
20	264.5 ± 6.1	261.45	255.80	276.26
25	264.6 ± 6.0	256.04	276.16	261.60
35	273.1 ± 6.4	263.97	269.90	285.43
45	273.3 ± 6.5	285.83	270.05	264.02
65	279.4 ± 7.1	275.85	269.27	293.08
85	281.0 ± 7.2	270.73	294.87	277.40
185	365.2 ± 9.8	360.30	351.21	384.09
305	406.3 ± 10.5	401.05	391.32	426.53
1296	421.5 ± 11.2	405.52	443.08	415.90
1526	424.2 ± 11.4	446.17	418.50	407.93
7196	505.3 ± 13.7	485.75	531.70	498.45
11,726	574.3 ± 15.6	604.36	566.50	552.04
17,026	2616.0 ± 42.3	2594.85	2555.64	2697.51

**Table 5:** Kinetic parameters obtained from the pseudo-first-order (PFO) and Schott pseudo-second-order (PSO) models for the initial swelling stage of the CMC/ECH hydrogel.

Hydrogel	Pseudo-First Order Kinetics			Pseudo-Second Order Kinetics		
Q_exp_ (%)	R^2^	K_1_	Q_cal_	R^2^	K_2_	Q_cal_
2616	0.5757	0.0019	2467.84	0.9963	0.00211	285.71

**Table 6:** Time-dependent water loss and Water Retention Data (mean ± SD, *n* = 3).

Time (min)	Net Weight (g)	Water Loss (%) (Mean ± SD)	Water Loss (%) Trial		
			1	2	3
0	60.390	0.0000 ± 0.0000	0.0000	0.0000	0.0000
5	60.182	0.3444 ± 0.012	0.3384	0.3273	0.3675
10	59.956	0.7187 ± 0.021	0.6887	0.7592	0.7082
15	59.746	1.0664 ± 0.033	1.1300	1.0499	1.0193
20	59.542	1.4042 ± 0.045	1.3817	1.3400	1.4909
25	59.282	1.8347 ± 0.058	1.8057	1.9465	1.7519
30	59.075	2.1775 ± 0.069	2.3105	2.0790	2.1430
40	58.704	2.7919 ± 0.091	2.7464	2.6620	2.9673
50	58.288	3.4807 ± 0.113	3.4242	3.6985	3.3194
70	57.599	4.6216 ± 0.150	4.9106	4.5466	4.4076
90	56.904	5.7725 ± 0.187	5.6790	6.1328	5.5057
110	56.229	6.8902 ± 0.223	6.7787	7.3199	6.5720
130	55.483	8.1255 ± 0.263	7.9940	7.7502	8.6323
170	54.318	10.0546 ± 0.325	9.5908	10.6809	9.8921
240	53.110	12.0550 ± 0.390	12.8065	11.8600	11.4985
270	51.465	14.7789 ± 0.478	14.5399	14.0968	15.7000
330	49.694	17.7115 ± 0.573	17.4250	18.8157	16.8938
390	47.746	20.9372 ± 0.678	20.5982	19.9697	22.2437

**Table 7:** Mean remaining mass and degradation rate of the hydrogel over time in the two carrier sets (mean ± SD, *n* = 3).

Time (Days)	Perforated Plastic Bag/Perforated Plastic Sack		Muslin/Cheesecloth (Cheesecloth Fabric)	
	Remaining Mass (g)	Degradation (%)/Mass Loss (%)	Remaining Mass (g)	Degradation (%)/Mass Loss (%)
0	0.5032 ± 0.0010	0.00 ± 0.00	0.5031 ± 0.009	0.00 ± 0.00
7	0.4840 ± 0.0145	3.81 ± 2.73	0.4268 ± 0.005	15.17 ± 0.915
14	0.4103 ± 0.059	18.45 ± 1.07	0.3404 ± 0.003	32.23 ± 0.630
21	0.3277 ± 0.0322	34.88 ± 0.51	0.2856 ± 0.005	43.24 ± 1.170
28	0.2956± 0.0112	41.25 ± 0.25	0.2320 ± 0.004	53.88 ± 0.806

## Data Availability

The data presented in this study are available in the article.
